# Gallium (III) Complexes with 5-Bromosalicylaldehyde Benzoylhydrazones: In Silico Studies and In Vitro Cytotoxic Activity

**DOI:** 10.3390/molecules27175493

**Published:** 2022-08-26

**Authors:** Boryana Nikolova-Mladenova, Silvia Angelova, Georgi Momekov

**Affiliations:** 1 Department of Chemistry, Faculty of Pharmacy, Medical University of Sofia, 2 Dunav Str., 1000 Sofia, Bulgaria; 2 Institute of Optical Materials and Technologies “Acad. Jordan Malinovski”, Bulgarian Academy of Sciences, 1113 Sofia, Bulgaria; 3 Department of Pharmacology, Pharmacotherapy and Toxicology, Faculty of Pharmacy, Medical University of Sofia, 2 Dunav Str., 1000 Sofia, Bulgaria

**Keywords:** 5-bromosalicylaldehyde, hydrazones, gallium (III) complexes, DFT calculations, cytotoxic activity

## Abstract

Gallium (III) complexes with the ligands 5-bromosalicylaldehyde-4-hydroxybenzoylhydrazone and 5-bromosalicylaldehyde isonicotinoylhydrazone were synthesized to receive compounds with improved antiproliferative action. Compounds were characterized by elemental analysis, IR, and NMR spectroscopy. Density functional theory calculations with Becke’s 3-parameter hybrid functional and 6-31+G(d,p) basis set were carried out to investigate the structural features of the ligands and Ga(III) complexes. Cytotoxic screening by MTT-dye reduction assay was carried out using cisplatin and melphalan as reference cytotoxic agents. A general formula [Ga(HL)_2_]NO_3_ for the complexes obtained was suggested. The complexes are mononuclear with the Ga(III) ions being surrounded by two ligands. The ligands acted as monoanionic tridentate (ONO) donor molecules. The analysis revealed coordination binding through deprotonated phenolic-oxygen, azomethine-nitrogen, and amide-oxygen atoms. The bioassay demonstrated that all compounds exhibited concentration-dependent antiproliferative activity at low micromolar concentrations against the acute myeloid leukemia HL-60 and T-cell leukemia SKW-3 cell lines. IC_50_ values of 5-bromo-derivative ligands and gallium (III) complexes are lower than those of cisplatin and much lower than these of melphalan. The coordination to gallium (III) additionally increased the cytotoxicity compared to the metal-free hydrazones.

## 1. Introduction

**Figure 1:**
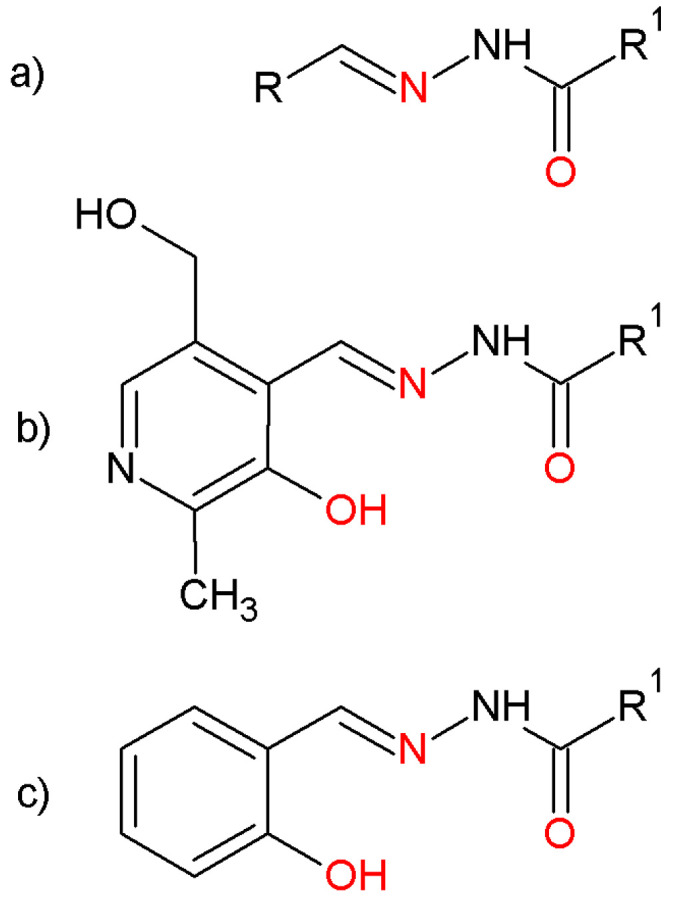
Structure and donor atoms of (**a**) aroylhydrazones, (**b**) hydrazones derived by pyridoxal, and (**c**) hydrazones derived by salicylaldehyde.

Aroylhydrazones of the type R-CH=N-NH-CO-R′, derived by the condensation of aromatic aldehydes and acyl hydrazides, form a series of bioactive compounds. They can act as bidentate ligands by utilizing the available azomethine nitrogen and amide oxygen donor sites (Figure 1a). Substituents in aldehydes or hydrazides can improve the coordination properties and make them versatile ligands with various coordination modes. Especially interesting hydrazones were synthesized by pyridoxal (3-hydroxy-5-(hydroxymethyl)-2-methylpyridine-4-carbaldehyde) (Figure 1b) and salicylaldehyde (2-hydroxybenzaldehyde) (Figure 1c) [1,2]. The additional hydroxy group in the aldehyde part increases the number of donor atoms and enhances the coordination ability of the ligands. Various derivative aldehydes have been used in order to discover new biologically active compounds [3,4,5,6,7]. Pyridoxal isonicotinyl hydrazone (PIH), salicylaldehyde benzoyl hydrazone (SBH), and their analogues are used as chelating agents for reducing the toxic effects of iron-overload diseases, such as β-thalassemia [4,8,9,10,11,12]. As the iron is crucial for the vitality of the cells, the compounds additionally exhibit an expressed cytotoxic activity and inhibit the proliferation of a variety of malignant cells in vitro [3,4,5,6,13,14]. Furthermore, coordination to metal ions may enhance the bioactivity of organic compounds and could promote desirable properties. The gallium(III) complex with pyridoxal isonicotinoyl hydrazone (Ga-PIH) is cytotoxic to solid-tumor and lymphoma cell lines [15].

Ga(III) ion possesses a substantial chemical similarity with Fe(III), possessing comparable values for ionic radius, ionization potential, and electron affinities [15,16,17]. This contributes to its biological activity and allows its interaction with important iron proteins under physiological conditions. Ga(III) is supposed to be able to substitute Fe(III) in ribonucleoside diphosphate reductase enzyme (RDR), which is involved in the conversion of ribonucleotides to deoxyribonucleotides during DNA synthesis. Gallium is thus regarded as an inhibitor of DNA synthesis and cell proliferation [15,18]. Ga(III) nitrate and Ga(III) chloride were discovered to have antitumor activity by themselves [15,18,19,20]. Gallium coordination with polydentate organic ligands has been introduced to avoid hydrolysis as the Ga(III) complexes demonstrate several advantages over gallium salts regarding oral bioavailability, hydrolytic stability, and membrane-penetration ability [21,22,23]. Gallium ions are expected to form analogous complexes with the same coordination numbers and geometry as iron(III). Encouraging compounds are found to be tris(8-quinolato)gallium(III) and tris(maltolato)gallium(III), which have entered the clinical trial phase and which are being evaluated as potential oral antitumor agents [24]. Gallium complexes with thiosemicarbazone ligands have been extensively investigated [25,26], and the cytotoxic activity of the ligands has been enhanced by the formation of metal complexes [27,28]. In the search for Ga(III) bioactive compounds, complexes with salicylaldehyde semicarbazone ligands have also been synthesized and explored for cytotoxicity [29,30,31].

Preparing Ga(III) complexes with semicarbazones, thiosemicarbazones and pyridoxal, and salicylaldehyde-derived hydrazones is based on the circumstance that both the ligands and metal center have antiproliferative properties and are aimed at the same molecular target [32]. In view of these observations, new gallium (III) complexes with salicylaldehyde-based hydrazones were synthesized. The ligands were designed with 5-bromosalicylaldehyde as the presence of a bromine atom in the molecules of some hydrazones greatly increases the bioactivity of the compounds [31,33]. All compounds were characterized by elemental analysis, IR spectroscopy, and NMR spectroscopy. DFT calculations were carried out to optimize the geometry of the ligands and metal complexes and to examine the coordination ability of bromo-hydrazones and the structural features of the complexes. The cytotoxic properties of the ligands and complexes were tested by MTT-dye reduction assays on HL-60 and SKW-3 human leukemic cell lines.

## 2. Results and Discussion

### 2.1. Preparation and Characterization

**Scheme 1:**
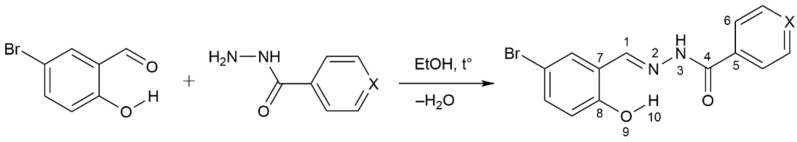
Synthesis of the 5-bromo-substituted ligands H_2_L^1^ (X = C-OH) and H_2_L^2^ (X = N).

The ligands 5-bromosalicylaldehyde-4-hydroxybenzoylhydrazone (H_2_L^1^) and 5-bromosalicylaldehyde isonicotinoylhydrazone (H_2_L^2^) were synthesized using the Schiff base condensation reaction in ethanol between 5-bromosalicylaldehyde and appropriate hydrazides-4-hydroxybenzhydrazide and isonicotinic acid hydrazide according to Scheme 1.

The ligands were obtained in excellent yields and were characterized with various analytical and spectroscopic techniques. Their crystal structures were determined by single-crystal X-ray diffraction analysis [34,35,36]. The melting points and the elemental analysis of the hydrazones, along with their IR, ^1^H NMR, and ^13^C NMR data, are given in the Section 3. The results are in good agreement with earlier, published data [36]. The elemental analysis confirms the molecular formulas of the compounds. IR spectra of the hydrazones (Supplementary Materials Figures S1 and S4) show an intensive band around 1609–1617 cm^−1^ assigned to the azomethine group C=N, which proves the condensation between the aldehyde group of 5-bromosalicylaldehyde and the amino group of hydrazides upon formation of the Schiff base. The medium-intensity peak around 3420–3520 cm^−1^ and the weak, broad band at 3171–3303 cm^−1^ were assigned to the phenolic hydroxyl group and NH group, respectively. The intensive, characteristic bands at 1660 and 1676 cm^−1^ in the spectra of the ligands were assigned to the frequency vibration of the carbonyl group C=O and suggest the existence of the ligands in keto form in a solid state. Another important band at 1589–1600 cm^−1^ was attributed to ν(C-NH). The hydrazones were further studied by their ^1^H NMR and ^13^C NMR spectra in DMSO-*d*_6_ (Supplementary Materials Figures S2, S3, S5 and S6). The ^1^H NMR spectra revealed the presence of the aromatic protons in the region of 6.88–8.80 ppm. Signals for the protons of the characteristic for the hydrazones azomethine group HC=N- were observed at 8.56 and 8.65 ppm, respectively, for H_2_L^1^ and H_2_L^2^. The broad singlets around 11.98 and 12.34 ppm were assigned to the protons of the hydroxyl group from the aldehyde ring. ^13^C NMR spectra demonstrated signals corresponding to the carbon atoms of azomethine group at 145.02 and 146.34 ppm, respectively, for H_2_L^1^ and H_2_L^2^. The peaks at 161.46 and 162.54 ppm were assigned to the C=O group.

**Scheme 2:**
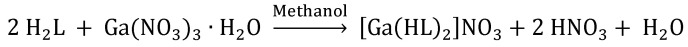
Synthesis of the gallium (III) complexes with the ligands H_2_L.

Two new gallium (III) complexes with the ligands H_2_L^1^ and H_2_L were also prepared by a direct reaction of the hydrazones and gallium (III) nitrate monohydrate in absolute methanol with good yields (Scheme 2).

Bis-(5-bromosalicylaldehyde-4-hydroxybenzoylhydrazone) gallium (III) nitrate (1) is a yellow solid and bis-(5-bromosalicylaldehyde isonicotinoylhydrazone) gallium (III) nitrate (2) is a dark-orange solid.

The complexes were characterized by elemental analysis as a basis for the determination of their empirical formulas. Experimental and calculated C, H and N values are in good agreement and reveal a 1:2 metal:ligand molar ratio in the complexes. Analyses suggest the following molecular formulas for the compounds: [Ga(C_14_H_10_N_2_BrO_3_)_2_]NO_3_ (1) and [Ga(C_13_H_9_N_3_BrO_2_)_2_]NO_3_ (2). The data on the elemental analysis of the gallium (III) complexes are summarized in the Section 3.

The IR spectra of the complexes (Supplementary Materials Figures S7 and S8) were compared with those of the ligands in order to determine the coordination sites involved in the chelation. The absence of characteristic bands due to ν(OH) in the IR spectra of the Ga(III) complexes supports the suggestion of the deprotonation of the ligands during the coordination and the displacement of a proton by the Ga(III) ion. A considerable negative shift was observed in the absorption frequency of the carbonyl group, showing coordination through the carbonyl-oxygen atom of the ligands in keto form. The bands observed at 1609 and 1617 cm^−1^ in the spectra of the ligands, which were attributed to azomethine C=N group, were shifted in the spectra of the complexes to lower wave numbers, indicating the involvement of the N-atom of the azomethine group in the complex formation. Absorptions at 1371 and 1354 cm^−1^ in the spectra of the complexes were attributed to the nitrate group as a counter-ion. The NH stretching vibrations in the free ligands’ spectra remained unaffected after complexation. This precluded the possibility of coordination through the imine nitrogen atom.

The comparison of the IR data of the Ga(III) complexes with those of the free ligands suggests that 5-bromosalicylaldehyde-4-hydroxybenzoylhydrazone and 5-bromosalicylaldehyde isonicotinoylhydrazone act as mono-anionic tridentate ligands, and they chelate Ga (III) ion through phenolic-oxygen, azomethine-nitrogen, and amide-oxygen atoms, forming a six-membered chelate ring.

**Figure 2:**
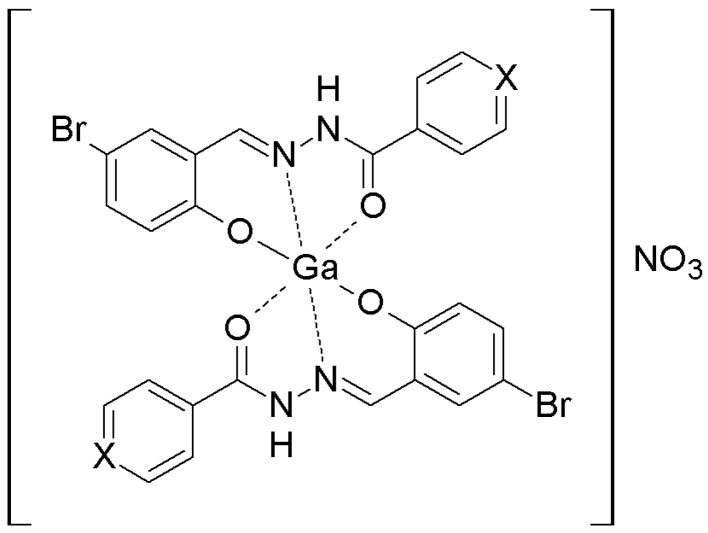
The suggested structure of Ga(III) Complexes 1 (X = C-OH) and 2 (X = N).

The ^1^H NMR data of the gallium (III) complexes were recorded in DMSO-*d*_6_. The results were interpreted based on the atom-labeling in Scheme 1. The absence of the signals at 11.99 ppm and 12.34 ppm for the proton of the phenolic OH groups in the ^1^H NMR spectra of the complexes indicated that the phenolic oxygen was coordinated to the Ga (III) ion after deprotonation. After complexation, the HC=N signals were shifted downfield to 8.65 ppm and 8.84 ppm in Complexes 1 and 2, respectively, indicating that the azomethine nitrogen coordinated to the Ga (III) ion. The resonance signals for the H_3_, H_4_, and H_6_ protons of the salicylaldehyde ring appeared at 6.90, 7.41 and 7.76 ppm for H_2_L^1^, and at 6.91, 7.44 and 7.83 ppm for H_2_L^2^. In the spectra of the complexes, these signals were shifted slightly to 6.84, 7.36 and 7.64 ppm for Complex 1, and to 6.81, 7.45 and 7.73 ppm for Complex 2. The signals for the protons from the hydrazide ring in the spectra of the complexes were also slightly shifted as compared to those of the ligands. The ^1^H NMR spectra of the Ga(III) complexes are consistent with the assumed structure derived from the IR spectra. Based on the above observations, the structure in Figure 2 for the Ga (III) complexes was suggested. A distorted octahedral geometry was experimentally proven for Ga-complexes with tridentate semicarbazone ligands [29].

### 2.2. Computational Study

**Figure 3:**
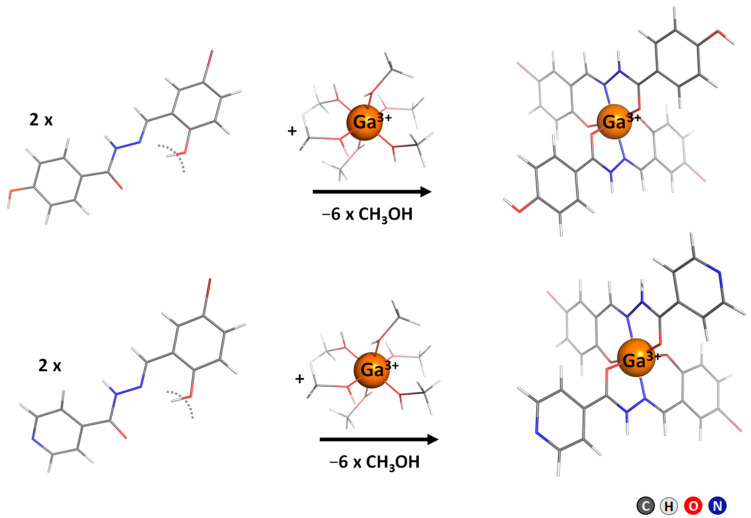
Optimized structures of the ligands and octahedral Ga(III) complexes.

The coordination of 5-bromosalicylaldehyde-4-hydroxybenzoylhydrazone and 5-bromosalicylaldehyde isonicotinoylhydrazone with Ga(III) ions in complexes with the proposed octahedral molecular structure (with metal bound to the oxygen atoms of phenolic and amide groups and to the azomethine nitrogen atom of each of the two ligands) was also investigated theoretically by means of DFT (density functional theory) calculations. The optimized geometry of the Ga-complex ions without a ${\mathrm{NO}}_{3}^{-}$ counter-ion is shown in Figure 3. Selected geometrical parameters for the studied ligands (neutral molecules and anions) and complexes are presented in Table 1.

The phenyl ring in the ligand structure is rotated about 27–28° (22.28° in neutral H_2_L^1^), so the free ligands are not planar. In the resulting complexes, the ligands are positioned almost perpendicularly to each other and are almost planarized in Complex 1; in Complex 2, only one of the ligands is almost planar. The calculated Ga-O and Ga-N distances are indicative of a stronger interaction of Ga centers with the deprotonated OH-groups. In all cases, the O9-Ga bonds (<2 Å) are shorter than the O11-Ga and N1-Ga bonds (>2 Å) (Table 1). The Ga-O and Ga-N bond distances agree well with the values measured for gallium(III) bis- and mono-tridentate complexes (1.881–2.004 Å and 1.961–2.180 Å for the Ga-O and Ga-N distances, respectively) [29]. There are no significant structural changes (in either the bond lengths or in the angles) in the ligands upon complex formation as the neutral ligands’ geometry is closer to the geometry of the respective complex. The Ga atom is positioned at an almost-equal distance from the N1 and O11 atoms in Complexes 1 and 2. Similar coordination behavior can be concluded for ligands H_2_L^1^ and H_2_L^2^ with Ga.

The results obtained for ΔG^1^ demonstrate that all of the complex formation reactions in the gas-phase are favorably characterized, with negative free energies that are quite large (Table 2). Solvation effects (treated implicitly), however, significantly attenuate the free energy gains in the gas-phase, but all of the values remain negative and quite large.

Ligands H_2_L^1^ and H_2_L^2^ are supposed to be able to displace ${\mathrm{NO}}_{3}^{-}$ from Ga(NO_3_)_3_ and to form Complexes 1 and 2: the calculated Gibbs energies in methanol for this reaction are −51.7 and −46.4 kcal mol^−1^ for the reaction with H_2_L^1^ and H_2_L^2^, respectively.

### 2.3. Pharmacology

The cytotoxic activity of the ligands and the respective gallium (III) complexes on the human leukemic cell lines HL-60 (human acute myeloid leukemia) and SKW-3 (T-cell leukemia) was studied using a standard MTT-dye reduction assay for cell viability. Throughout the screening investigation, the data on the ligands and Ga(III) complexes were compared with those on the clinically used antineoplastic drugs cisplatin and melphalan (2-amino-[4-bis(2-chloroethyl) amino] phenylpropanoic acid). 

All of the tested compounds exhibited concentration-dependent cytotoxic effects after the 72-h treatment of both HL-60 and SKW-3 cells, which allowed for the construction of sigmoidal dose-response curves and the calculation of the corresponding IC_50_ values. Each data point represents the arithmetic mean ± standard deviation (SD) of at least eight independent experiments. The IC_50_ values were calculated as concentrations of the tested compounds causing a 50% decrease in cell survival. The bioassay data are summarized in Table 3.

As evident from the results obtained, 5-bromo-substituted hydrazones and their gallium (III) complexes were highly active against leukemic cell lines and showed antiproliferative activity in the micromolar range. The ligand H_2_L^1^ produced an identical cytotoxic effect against the T-cell leukemic cells SKW-3 and the myeloid HL-60 cells, with IC_50_ values of 3.02–3.14 µmol/L, respectively. Its gallium (III) Complex 1 also exerted similar cytotoxic effects against both the cell lines with IC_50_ values of 1.14–1.31 µmol/L. The ligand H_2_L^2^ and its Complex 2 similarly displayed comparable cytotoxic activity on the leukemic cells. It is worth noting that Complex 2 exerted the most pronounced cytotoxic effects, and against T-cell leukemic cells SKW-3, the IC_50_ value was 0.54 µmol/L. Since the ligand H_2_L^2^ alone did not present equally good antiproliferative activity, the high cytotoxic effect was probably due to the complex as an entity. With a concentration of 50 µmol/L applied, all compounds caused a drastic decrease in the viability of the malignant cells of approximately 91–95%. IC_50_ values for the compounds are comparable but slightly less than that of cisplatin and much lower than that of melphalan. Complexation with gallium (III) ions led to the enhancement of the cytotoxic potencies of 5-bromo-substituted hydrazones according to the ratios of the respective IC_50_ values.

## 3. Materials and Methods

### 3.1. Preparation and Characterization 

All chemicals used were of analytical reagent grade. Gallium (III) nitrate monohydrate, 5-bromosalicylaldehyde, 4-hydroxybenzhydrazide, isonicotinic hydrazide, 96% ethanol, and 99.8% methanol were purchased from Merck (Darmstadt, Germany) and used without further purification. The carbon, nitrogen, and hydrogen contents of the compounds were determined by elemental analyses on a Euro EA 3000-Single, EuroVector SpA (Milan, Italy). The melting points were determined using a Buchi 535 apparatus (Flawil, Switzerland). The IR spectra in the range of 4000–400 cm^−1^ were recorded using KBr pallets on a Bruker Tensor 27 spectrophotometer (Ettlingen, Germany). The ^1^H and ^13^C NMR spectra were recorded on a Bruker Avance DRX 250 spectrophotometer (Rheinstetten, Germany) in DMSO-*d*_6_ as a solvent, using tetramethylsilane (TMS) as an internal standard. Chemical shifts (δ) were reported in parts per million (ppm); *J* values were given in Hz. Splitting patterns were indicated by the symbols: s (singlet), d (doublet), t (triplet), m (multiplet), and br (broad).

#### 3.1.1. Synthesis of the Ligands 5-Bromosalicylaldehyde-4-hydroxybenzoylhydrazone (H_2_L^1^) and 5-Bromosalicylaldehyde Isonicotinoylhydrazone (H_2_L^2^)

The ligands H_2_L^1^ and H_2_L^2^ were prepared according to previously published protocols [5,6,14]. In brief, a solution of 5-bromosalicylaldehyde (0.01 mol) in 96% ethanol (30 mL) was added to the solutions of 4-hydroxybenzhydrazide (0.01 mol) and isonicotinic hydrazide (0.01 mol) in 50% aqueous ethanol (80 mL). The mixtures were stirred for 30 min, and white precipitates were formed. The crude products were purified and recrystallized from ethanol to afford the crystals of the H_2_L^1^ and H_2_L^2^.

5-bromosalicylaldehyde-4-hydroxybenzoylhydrazone, H_2_L^1^

Yield: 85%; mp: 286–287 °C; Color: Yellow; IR (KBr, ν cm^−1^): 3420, 3500 (Ar-OH), 3303 (NH), 1660 (C=O), 1609 (C=N), 1589 (C-NH). ^1^H NMR (250 MHz, DMSO-*d*_6_) δ ppm: 6.88 (d, 2H, *J* = 8.75 Hz, ArH_hydrazide_), 6.90 (d, 1H, *J* = 8.75 Hz, ArH_aldehyde_), 7.41 (d, 1H, *J* = 8.75 Hz, ArH_aldehyde_), 7.76 (s, 1H, ArH_aldehyde_), 7.83 (d, 2H, *J* = 8.75 Hz, ArH_hydrazide_), 8.57 (s, 1H, CH=N), 10.16 (s, 1H, O-H_hydrazide_), 11.41 (s, 1H, N-H), 11.99 (s, 1H, O-H_aldehyde_); ^13^C NMR (250 MHz, DMSO-*d*_6_) δ ppm: 110.32, 115.07, 118.62, 121.30, 123.11, 129.78, 130.60, 133.27, 145.02 (CH=N), 156.37, 160.92, 162.54 (C=O). Calculated for C_14_H_11_N_2_O_3_Br: C, 50.17; H, 3.31; N, 8.36. Found: C, 49.92; H, 3.35; N, 8.40.

5-bromosalicylaldehyde isonicotinoylhydrazone, H_2_L^2^

Yield: 88%; mp: 259–260 °C; Color: Yellow; IR (KBr, ν cm^−1^): 3500 (Ar-OH), 3171 (N-H), 1676 (C=O), 1617 (C=N), 1600 (C-NH). ^1^H NMR (250 MHz, DMSO-*d*_6_) δ ppm: 6.91 (d, 1H, *J* = 8.75 Hz, ArH_aldehyde_), 7.44 (d, 1H, *J* = 8.75 Hz, ArH_aldehyde_), 7.83 (s, 1H, ArH_aldehyde_), 7.84 (d, 2H, *J* = 6 Hz, ArH_hydrazide_), 8.65 (s, 1H, CH=N), 8.80 (d, 2H, *J* = 6 Hz, ArH_hydrazide_), 11.12 (s, 1H, NH), 12.34 (br s, 1H, O-H). ^13^C NMR (250 MHz, DMSO-*d*_6_) δ ppm: 110.50, 118.67, 121.26, 121.46, 130.09, 133.85, 139.87, 146.34 (CH=N), 150.33, 156.42, 161.46 (C=O). Calculated for C_13_H_10_N_3_O_2_Br: C, 48.77; H, 3.15; N, 13.13. Found: C, 48.98; H, 3.28; N, 13.66.

#### 3.1.2. Synthesis of the Ga(III) Complexes [Ga(HL^1^)_2_]NO_3_ (1) and [Ga(HL^2^)_2_]NO_3_ (2)

The gallium (III) complexes were obtained by a reaction of Ga(NO_3_)_3_. H_2_O with the ligands in 1:2 metal-to-ligand molar ratio using the following general procedure: A solution of gallium nitrate monohydrate (0.5 mmol) in methanol (50 mL) was added to the respective solutions of the ligands (1 mmol) in methanol (100 mL), which resulted in the immediate precipitation of metal complexes. The mixtures were stirred for 1 h at room temperature to complete the reaction, and they were then allowed to stand undisturbed overnight. Fine crystals were collected after filtration, washed with methanol, and dried in a vacuum desiccator.

Bis-(5-bromosalicylaldehyde-4-hydroxybenzoylhydrazone) gallium (III) nitrate, [Ga(HL^1^)_2_]NO_3_ (1) 

Yield: 93%; Color: Yellow; IR (KBr, ν cm^−1^): 3303 (NH), 1604 (C=O), 1538 (C=N), 1371 (${\mathrm{NO}}_{3}^{-}$). ^1^H NMR (250 MHz, DMSO-*d*_6_) δ ppm: 6.76 (d, 2H, *J* = 8.75 Hz, ArH_hydrazide_), 6.84 (d, 1H, *J* = 8.75 Hz, ArH_aldehyde_), 7.36 (d, 1H, *J* = 8.75 Hz, ArH_aldehyde_), 7.64 (br s, 1H, ArH_aldehyde_), 7.91 (d, 2H, *J* = 8.75 Hz, ArH_hydrazide_), 8.65 (s, 1H, CH=N), 10.03 (s, 1H, O-H_hydrazide_). Calculated for [Ga(C_14_H_10_N_2_O_3_Br)_2_]NO_3_: C, 42.04; H, 2.52; N, 8.75. Found: C, 42.18; H, 2.83; N, 8.70.

Bis-(5-bromosalicylaldehyde isonicotinoylhydrazone) gallium (III) nitrate, [Ga(HL^2^)_2_]NO_3_ (2)

Yield: 96%; Color: Dark orange; IR (KBr, ν cm^−1^): 3171 (N-H), 1609 (C=O), 1540 (C=N), 1354 (${\mathrm{NO}}_{3}^{-}$). ^1^H NMR (250 MHz, DMSO-*d*_6_) δ ppm: 6.81 (d, 1H, *J* = 8.75 Hz, ArH_aldehyde_), 7.45 (d, 1H, *J* = 8.75 Hz, ArH_aldehyde_), 7.73 (s, 1H, ArH_aldehyde_), 8.02 (d, 2H, *J* = 6 Hz, ArH_hydrazide_), 8.84 (s, 1H, CH=N), 8.88 (d, 2H, *J* = 6 Hz, ArH_hydrazide_). Calculated for [Ga(C_13_H_9_N_3_O_2_Br)_2_]NO_3_: C, 40.56; H, 2.36; N, 12.73. Found: C, 40.83; H, 2.72; N, 13.12.

### 3.2. Computational Details

The B3LYP/31+G(d,p) (Becke’s three-parameter hybrid exchange-correlation functional [37] with diffuse function augmented double-ζ polarized basis set) method was used to optimize the geometry of each ligand and its metal complex. Bond distances and angles in 5-bromosalicylaldehyde benzoylhydrazone were used to verify the proper selection of the computational level: the B3LYP/31+G(d,p) level of theory reliably reproduces the geometry of the crystal 5-bromosalicylaldehyde benzoylhydrazones [34,35,38] (present work; see the Supplementary Data). All of the geometries were optimized without symmetry restrictions by gradient procedure. Local minima were verified by establishing that all vibrational frequencies were real. The vibrational frequencies were used to compute the thermal energies, E_th_, including the zero-point energy, and entropies, S. The differences, ΔE_el_, ΔE_th_, PΔV (work term), and ΔS, between the products and reactants were used to evaluate the gas-phase free energy of the complex-formation, ΔG^1^, at T = 298.15 K, according to Equation (1):

ΔG^1^ = ΔE_el_ + ΔE_th_ + PΔV − TΔS (1)

All of the species used for the ΔG^1^ calculation are at their optimized geometries. The fully optimized structure of each molecule/complex in the gas-phase was subjected to a single-point SMD (density-based solvation model [39]) calculation in methanol (with dielectric constant e = 33). The difference between the gas-phase and SMD energies yielded the solvation energy of the reactants (ions)/products (complexes). These solvation free energies were used to calculate the free energy of the complex-formation in a condensed medium (methanol) according to Equation (2):

∆G^ε^ =∆G^1^ + ∆G_solv_^ε^ (Products) − ∆G_solv_^ε^ (Reactants) (2)

A negative ΔG^ε^ implies a thermodynamically favorable complex-formation, whereas a positive value implies an unfavorable one. The ΔG values were corrected with the basis set superposition error (BSSE) computed for the complexes using the counterpoise method [40]. All calculations were performed using Gaussian 09 [41]. PyMOL molecular graphics software was used for generating the molecular graphics images [42].

### 3.3. Cell Lines and Culture Conditions

The cell lines used in this study, namely HL-60 and SKW-3, were purchased from the German Collection of Microorganisms and Cell Cultures. HL-60 is a human acute myeloid leukemia cell line, established from the peripheral blood of a 35-year-old woman with acute myeloid leukemia in 1976. SKW-3 is a human T-cell leukemia cell line, originally described as having been established from the peripheral blood of a 61-year-old man with T-cell chronic lymphocytic leukemia in 1977.

The cells were grown in cell-culture flasks at 37 °C in an incubator, “BB 16-Function Line” Heraeus, with a humidified atmosphere and 5% carbon dioxide under standard conditions-RPMI 1640 liquid media supplemented with 10% fetal bovine serum (FBS) and 2 mM L-glutamine. The logarithmic growth phase of the cell cultures was maintained by supplementing with fresh media, two or three times per week.

### 3.4. Cytotoxicity Assessment (MTT-Dye Reduction Assay)

The MTT [3-(4,5-dimethylthiazol-2-yl)-2,5-diphenyltetrazolium bromide] assay was used for the determination of cell viability and proliferation. The method is based on the conversion of the yellow tetrazolium compound MTT to water-insoluble, violet formazan crystals by dehydrogenases occurring in the mitochondria of living cells as described by Mossman [43]. Briefly, exponentially proliferating cells were seeded in 96-well, flat-bottomed microplates (100 μL/well) at a density of 1 × 10^5^ cells per mL and then exposed to various concentrations of the tested substances for 72 h following a 24-h incubation period at 37 °C. At least eight wells were used for each concentration. Mixed overnight with the tested compounds, aliquots of 10 μL MTT solution were added to each well. The microplates were then incubated at 37 °C for 4 h. The formed MTT-formazan crystals were dissolved with 100 μL/well 5% HCOOH in 2-propanol, and the absorption was measured at 580 nm using a microplate reader (Labexim LMR-1).

### 3.5. Data Processing and Statistics

Normalized cell-survival data were calculated as a percentage of the untreated control (set at 100 percent viability). The Student’s t-test was used to analyze the biological data, and values of *p* ≤ 0.05 were considered statistically significant. Furthermore, IC_50_ values were calculated from concentration-response curves using non-linear regression analysis.

## 4. Conclusions

In our search for bioactive compounds, 5-bromosalicylaldehyde-4-hydroxybenzoylhydrazone and 5-bromosalicylaldehyde isonicotinoylhydrazone ligands and their gallium (III) complexes were synthesized and characterized by elemental analyses and spectral investigations. The 5-bromohydrazones acted as monoanionic tridentate ligands, coordinating to the gallium ion through ONO donor sites, thus forming stable, six-membered chelates. The analytical data suggest a general formula [Ga(HL)_2_]NO_3_ for the complexes. The molecular geometries of the ligands and gallium (III) complexes were studied at the B3LYP/6-31+G(d,p) level of theory. Selected geometrical parameters for the studied ligands and complexes were compared, and the structural changes in the ligands upon complex-formation have been discussed. The pharmacological activity of the ligands and Ga(III) complexes was studied using standard MTT-dye reduction assays for cell viability. All compounds exerted concentration-dependent, antiproliferative effects after a 72-h exposure. The preliminary cytotoxicity screening revealed that the investigated 5-bromosalicylaldehyde derived hydrazones and gallium complexes showed high antiproliferative activity and induced a 50% inhibition of the cell viability of HL-60 and SKW-3 cells at low micromolar concentrations. The IC_50_ values of gallium complexes were lower, but they were comparable to those of cisplatin and much lower than those of melphalan. Coordination to gallium (III) ions led, in general, to the enhancement of the cytotoxic potencies of 5-bromo-substituted hydrazones, according to the ratios of the respective IC_50_ values.

## Figures and Tables

**Table 1:** Selected B3LYP/6-31+G(d,p) calculated parameters for the ligands and Ga(III) complexes.

Parameter	H_2_L^1^	H_2_L^2^	HL^1^ (Anion)	HL^2^ (Anion)	1	2
Dipole moment, *μ* [D]	5.46	3.49	12.23	8.75	11.73	5.96
Bond length [Å]						
C1-N2	1.290	1.290	1.299	1.301	1.304	1.307
N2-N3	1.357	1.359	1.381	1.379	1.375	1.376
N3-C4	1.391	1.385	1.366	1.363	1.359	1.351
C4-C5	1.494	1.504	1.512	1.514	1.462	1.477
C4-O11	1.223	1.221	1.230	1.230	1.263	1.259
C5-C6	1.406	1.401	1.405	1.402	1.411	1.440
C1-C7	1.453	1.452	1.439	1.436	1.424	1.411
C7-C8	1.422	1.422	1.472	1.473	1.437	1.429
C8-O9	1.345	1.345	1.253	1.252	1.306	1.307
O9-H10/O9-Ga	0.987	0.986	-	-	1.900	1.897
N1-Ga	-	-	-	-	2.025	2.024
O11-Ga	-	-	-	-	2.025	2.033
Angle [◦]						
C1-N2-N3	119.06	118.76	112.52	112.45	120.25	120.11
N2-N3-C4	119.85	119.87	123.69	123.58	116.40	116.07
N3-C4-O11	121.77	122.78	125.28	125.85	117.56	118.30
N3-C4-C5	115.18	114.72	114.14	113.92	120.33	119.95
C5-C4-O11	123.05	122.50	120.58	120.23	122.11	121.75
C1-C7-C8	121.88	121.92	124.03	123.93	122.60	122.66
Dihedral angle [◦]						
N2-C1-C7-C8	0.66	0.45	−0.44	−0.08	0.28	0.32
N3-C4-C5-C6	−22.28	−27.34	−27.59	−27.68	4.12	3.41/16.31

**Table 2:** Calculated Gibbs energies for complex-formation in the gas-phase, ∆G^1^, and in a methanol environment, ∆G^33^, for the complex-formation reaction with methanol solvated Ga cation (2xLigand + Ga(CH_3_OH)_6_→Ga-complex + 6xCH_3_OH) (in kcal∙mol^−1^).

Complex Ion	∆G^1^	∆G^33^
1	−489.7	−71.2
2	−471.5	−65.9

**Table 3:** Cytotoxic activity of the tested ligands and Ga (III) complexes after 72-h treatment.

Compound	IC_50_ [µmol/L] ± SD	
	HL-60 ^a^	SKW-3 ^b^
H_2_L^1^	3.14 ± 1.1	3.02 ± 1.05
H_2_L^2^	4.13 ± 1.2	2.53 ± 1.06
1	1.31 ± 0.7	1.14 ± 0.6
2	2.45 ± 1.0	0.54 ± 0.2
cisplatin	8.70 ± 2.4	11.40 ± 2.1
melphalan	18.50 ± 2.1	31.30 ± 2.9

^a^ Acute myeloid leukemia, ^b^ T-cell leukemia.

## Data Availability

Not applicable.

## Supplementary Materials

The following supporting information can be downloaded at: https://www.mdpi.com/article/10.3390/molecules27175493/s1, Table S1: Selected bond lengths (Ǻ) and angles (°) for 5-bromosalicylaldehyde derivatives; Figures S1–S8: IR and NMR spectra of the ligands and their complexes.

## Sample Availability

Samples of the investigated compounds are available from the authors.
