# Supplementary material for: Gallium (III) Complexes with 5-Bromosalicylaldehyde Benzoylhydrazones: In Silico Studies and In Vitro Cytotoxic Activity
Source: Molecules. 2022 Aug 26;27(17):5493. doi: 10.3390/molecules27175493 (PMC9457627; doi:10.3390/molecules27175493)
Supplement: Supplementary file 1 [file molecules-27-05493-s001.zip › molecules-1861853-supplementary.pdf]

Supplementary data

The structures of 5-bromosalicylaldehyde benzoylhydrazone (**5BrSBH**), 5-bromosalicylaldehyde-4-hydroxybenzoylhydrazone (H_2L^1), and 5-bromosalicylaldehyde isonicotinoylhydrazone (H_2L^2) are determined experimentally by X-ray diffraction [S1-S3]. In order to predict the molecular structures of the ligands, quantum-chemical calculations were carried out. The structural parameters of the hydrazones (5-bromosalicylaldehyde benzoylhydrazone **5BrSBH**, 5-bromosalicylaldehyde-4-hydroxybenzoyl-hydrazone H_2L^1, and 5-bromosalicylaldehyde isonicotinoylhydrazone H_2L^2) were computed at the B3LYP level of theory using a 6-31+G (d, p) basis set. The calculated bond lengths and angles were compared with the experimental crystallographic data for **5BrSBH**, H_2L^1, and H_2L^2. As listed in Table S1, the calculated bond values are in good agreement with the experimental electron diffraction data. A small difference appears between the measured and the calculated O-H bond, due to the possible involvement of a hydroxyl group in an intramolecular O-H…N hydrogen bond. The values of the calculated angles perfectly match the experimentally measured ones. Therefore, it can be concluded that the substitutions in the hydrazide aromatic nuclei have not influenced the geometries of the new derivatives, and all compounds have similar structures.

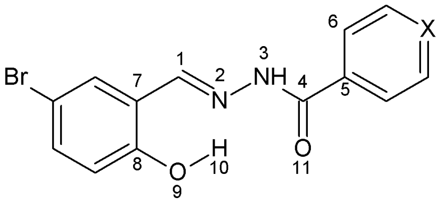

5BrSBH: X=CH
H_2L^1**:** X=C-OH
H_2L^2**:** X=N

Table S1. Selected bond lengths (Å) and angles (°) for 5-bromosalicylaldehyde derivatives.

Selected geometric parameters (Å, °)	**5BrSBH** exp. [S1]	**5BrSBH** calc.	H_2L^1exp. [S2]	H_2L^1calc.	H_2L^2exp. [S3]	H_2L^2calc.
C1-N2	1.275	1.290	1.288	1.290	1.270	1.290
N2-N3	1.362	1.357	1.365	1.357	1.369	1.359
N3-C4	1.363	1.389	1.364	1.391	1.353	1.384
C4-C5	1.479	1.499	1.483	1.494	1.507	1.504
C4-O11	1.223	1.221	1.228	1.222	1.204	1.221

C1-C7	1.456	1.452	1.458	1.453	1.450	1.451
C8-O9	1.345	1.345	1.365	1.345	1.351	1.345
O9-H10	0.820	0.987	0.764	0.987	0.820	0.986
C1-N2-N3	119.0	119.0	117.8	119.0	119.7	118.8
N2-N3-C4	118.5	119.8	118.8	119.8	115.1	119.8
N3-C4-O11	121.3	122.1	120.7	121.7	122.3	122.7
N3-C4-C5	115.7	114.9	117.8	115.2	116.1	114.7
C5-C4-O11	123.1	123.0	121.6	123.0	121.6	122.4
C1-C7-C8	122.0	121.9	122.9	121.9	122.0	121.9

[S1] H.-Y. Liu, H.-Y. Wang, F. Gao, Z.-S. Lu. D.-Z. Niu, 5-Bromosalicylaldehyde benzoylhydrazone Acta Cryst. (2006). E62, o4495-o4496, doi:10.1107/S160053680603683X.

[S2] Z.-L. Jing, M. Yu, X. Chen, (E)-N′-(5-Bromo-2-hydroxybenzylidene)- 4-hydroxybenzohydrazide ethanol solvateActa Cryst. (2007). E63, o4902, doi:10.1107/S1600536807060667.

[S3] D.-S. Yang, N′-[1-(5-Bromo-2-hydroxyphenyl)methylidene]- isonicotinohydrazide, Acta Cryst. (2006). E62, o3792–o3793, doi:10.1107/S1600536806030765.

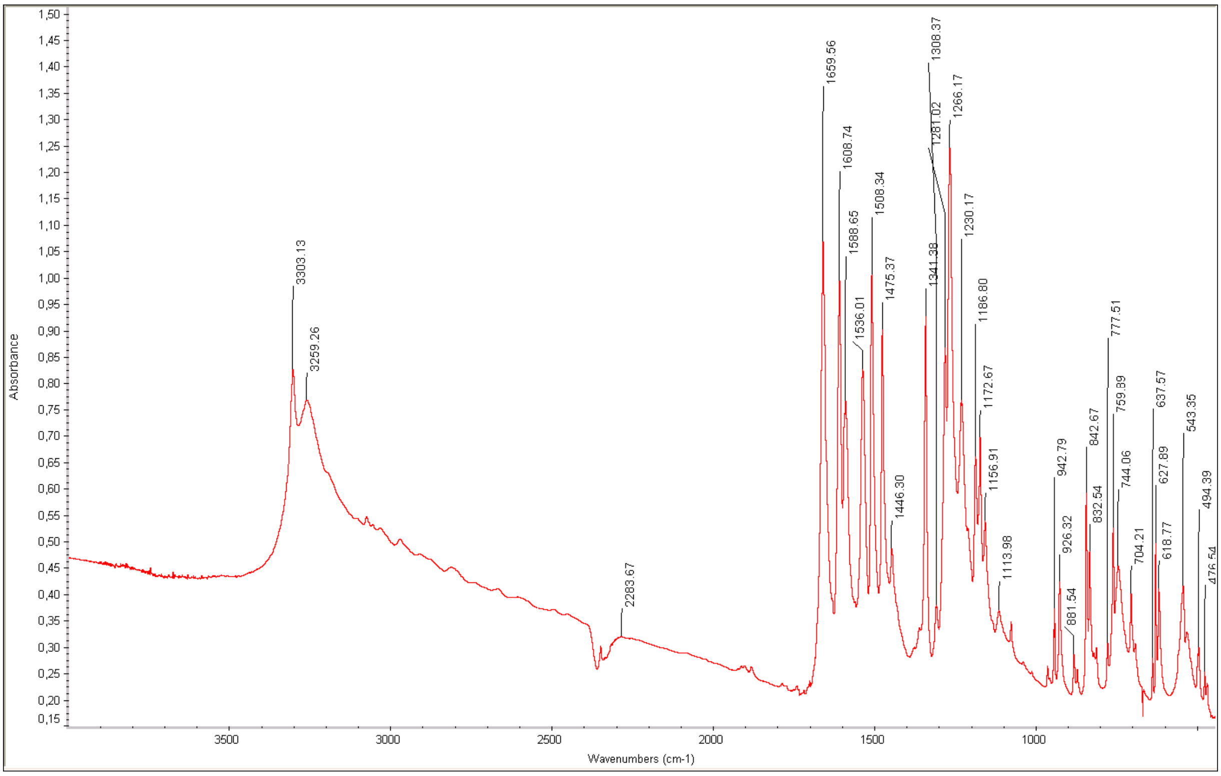

Figure S1. IR spectra of the ligand H_2L^1.

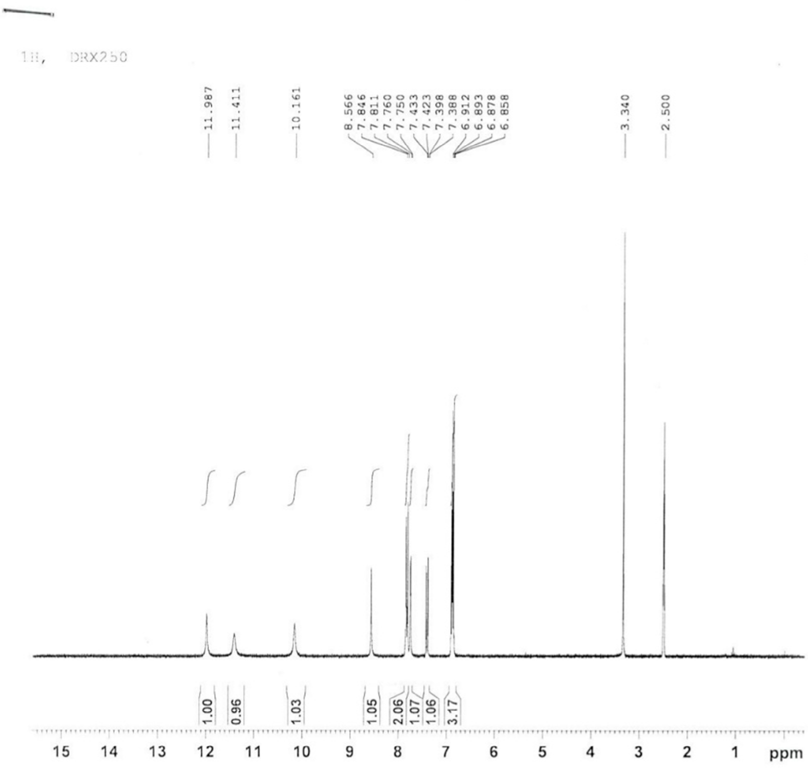

Figure S2. ^1H NMR spectra of the ligand H_2L^1.

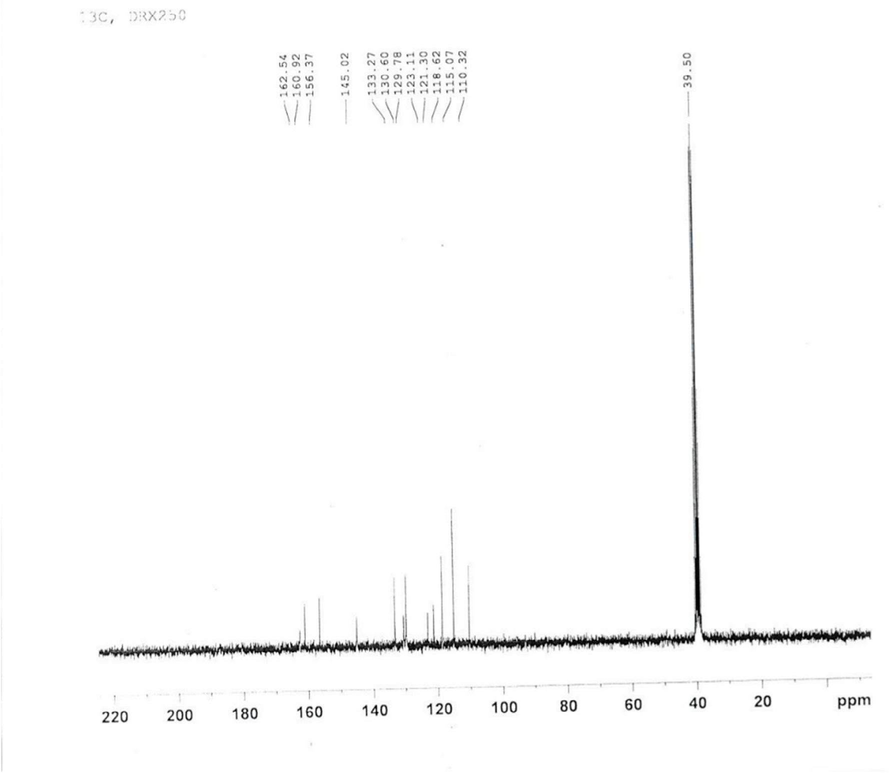

Figure S3. ^{13}C NMR spectra of the ligand H_2L^11.

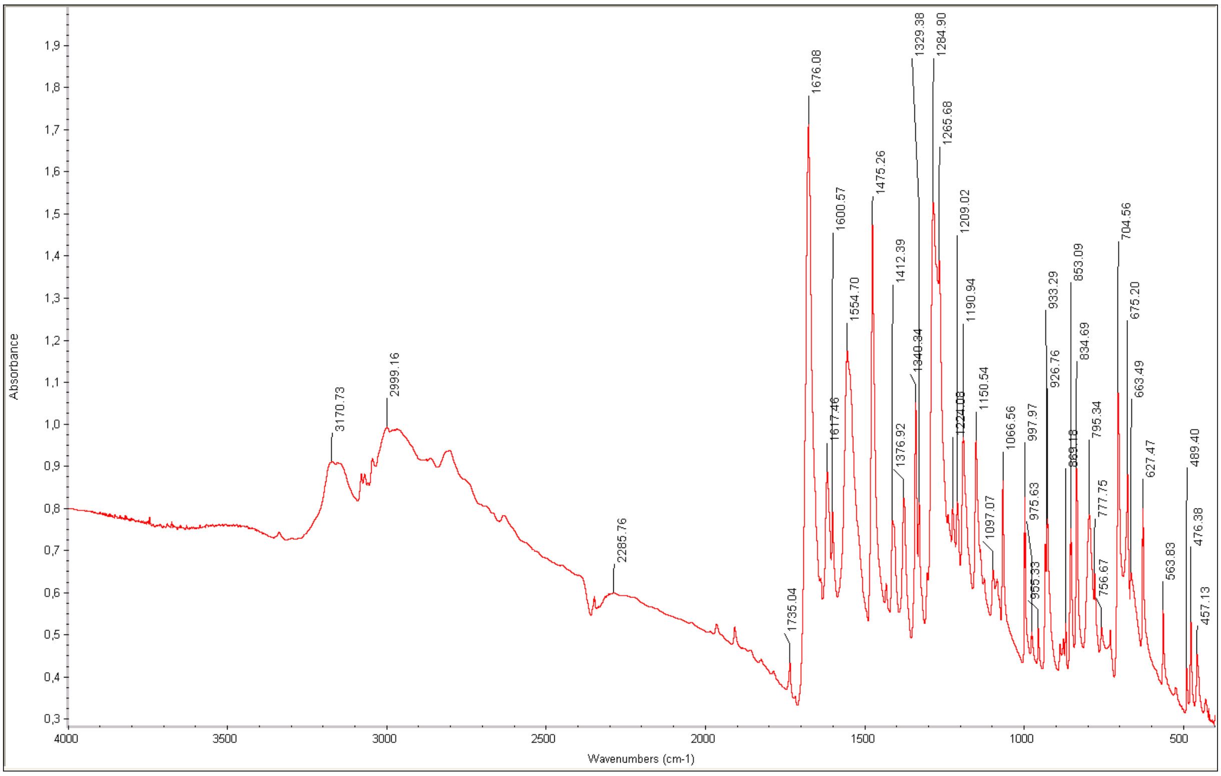

Figure S4. IR spectra of the ligand H_2L^2.

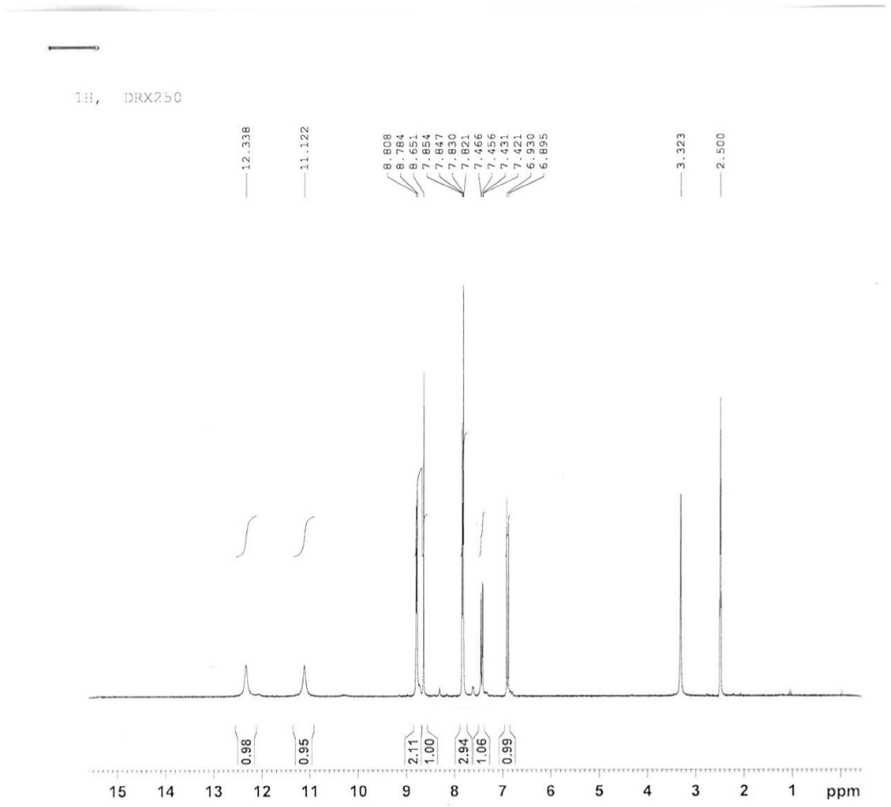

Figure S5. ^{1}H NMR spectra of the ligand H_2L^2.

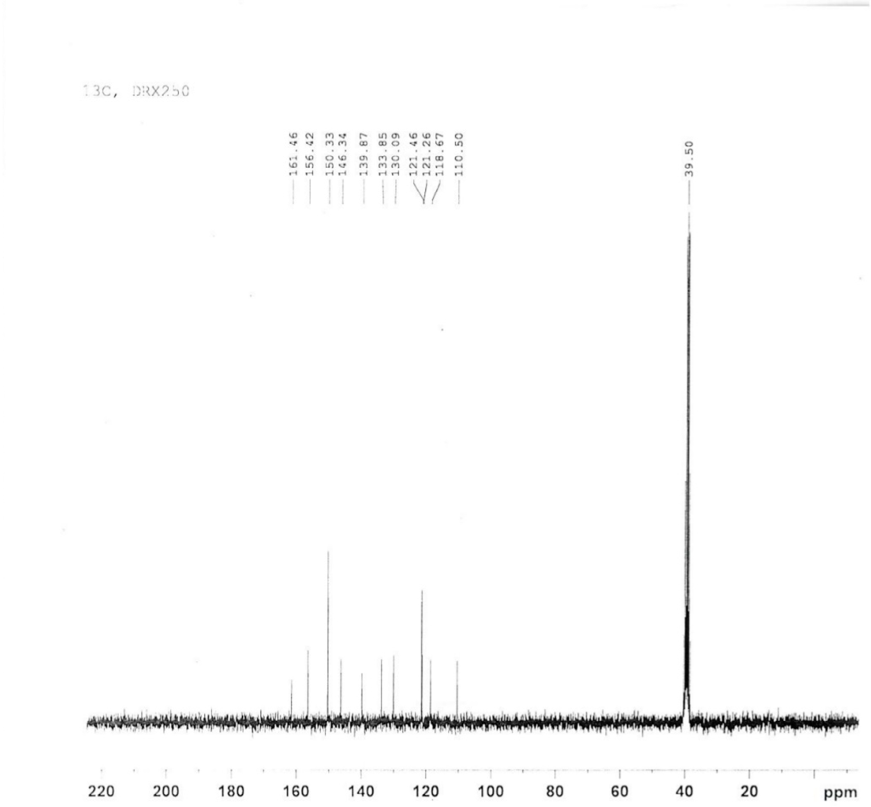

Figure S6. ^{13}C NMR spectra of the ligand H_2L^2.

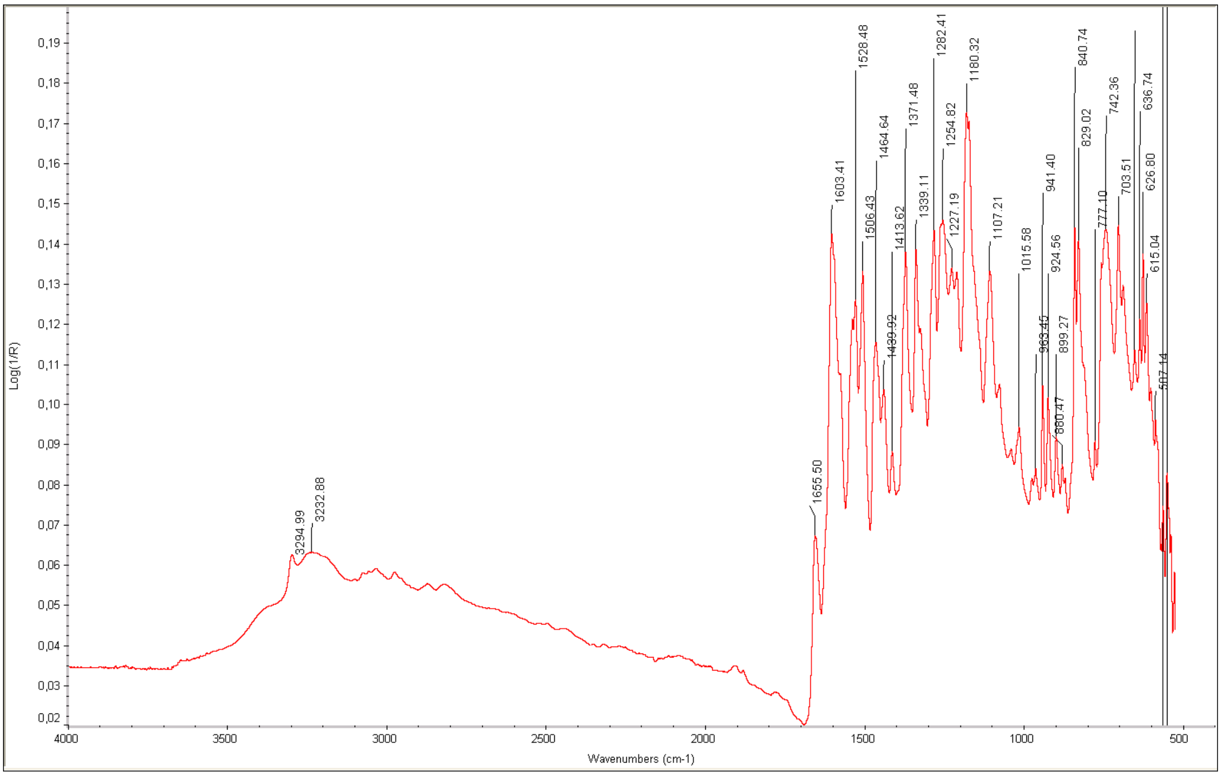

Figure S7. IR spectra of Complex 1.

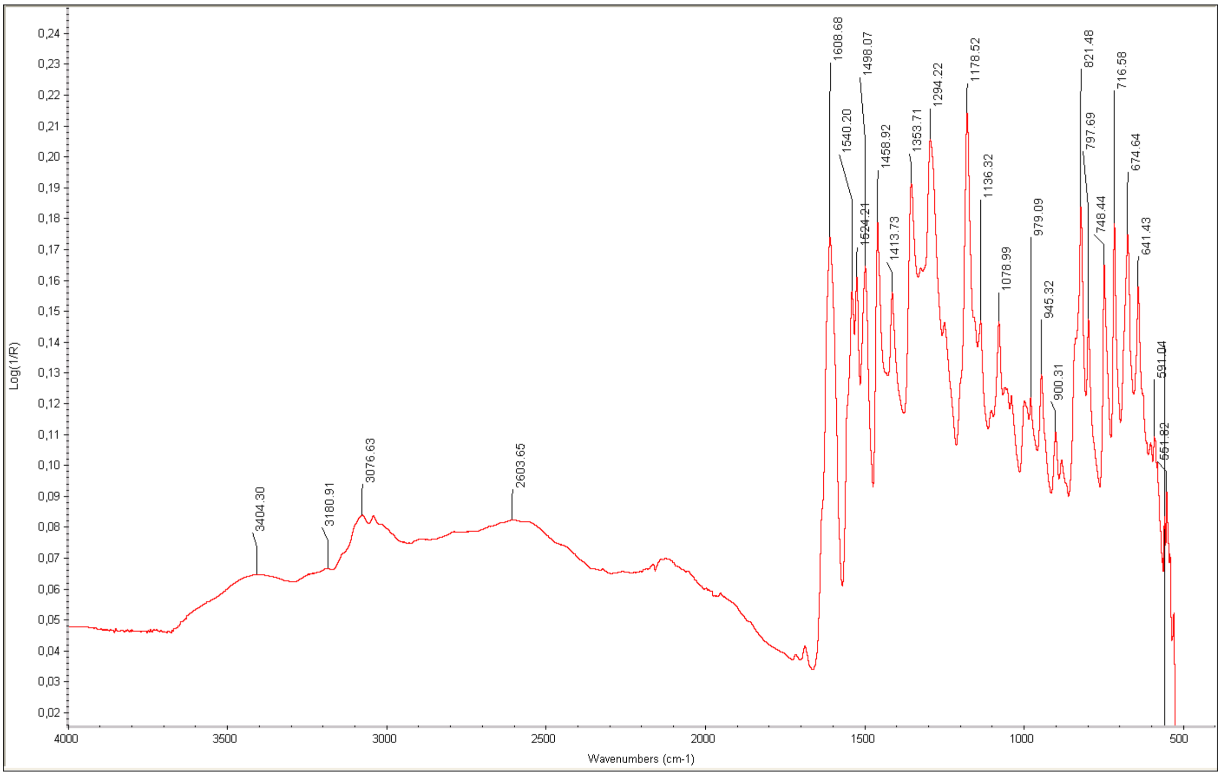

Figure S8. IR spectra of Complex 2.
